# Books and Magazines

**Published:** 1904-08

**Authors:** 


### Books and Magazines

PRACTICAL MEDICAL SERIES.—We have before us the following volumes of the Practical Medical Series: "General Medicine," by Billings and Salisbury (October, 1903); "General Surgery," by Murphy (November, 1903); "Eye, Ear, Nose, Throat," by Wood, Andrews, and Head (December, 1903); "Gynecology," by Dudley Healy (March, 1904); "Obstetrics," by DeLee (April, 1904); "General Medicine," by Billings (May, 1904).

It is needless to speak of the value of this series of books; their value to the practicing physician is too well known, but we desire to remind our readers that the arrangement of the books in several volumes enable those interested in special subjects to buy only the parts they desire.

This series of books is published by the Year Book Publishing Co., 40 Dearborn Street, Chicago. Price, $1 and $1.50 per volume.

---

PROGRESSIVE MEDICINE—A QUARTERLY DIGEST OF ADVANCES, DISCOVERIES AND IMPROVEMENTS IN THE MEDICAL AND SURGICAL SCIENCES. Edited by Hobart Hare, M. D., assisted by H. R. M. Landis. The March number is fresh from the press. Lea Brothers & Company, publishers.

The 1904 series is bound in paper and the book, formerly in cloth at $2.50 per volume, now sells for $1.50. There is much valuable information in it, and the reasonable price for which it sells will enable every physician to obtain it for his library.

---

THE MEDICAL AND SURGICAL REPORT of the Presbyterian Hospital in the city of New York is just out and it contains some very able papers, together with a number of fine illustrations. Dr. John Howland's paper on the "Pathological Anatomy of Shiga Bacillus Infection of the Intestines in Infants," won the Briddon medal. The paper is very interesting and instructive.

J. D.

---

THE DOCTOR'S RECREATION SERIES. Charles Wells Moulton, general editor. Published by the Saalsfield Publishing Company, Akron, O. Facts and fancies of interest to the doctor and his patient. Arranged by Porter Davis, M. D. Price per volume, cloth, $2.50; leather, $4. 352 pages.

We have received Volume I of the series. It is entitled "The Doctor's Leisure Hour." We are simply delighted with it. It is something new, and is bright, breezy and brief. It is the very thing for a tired doctor to read and leave in his waiting-room for his tired patients to dip into while waiting. Besides being interesting, the volume is ornamental. The one we have is bound in chocolate cloth, gilt and green, on thick, but very light, embossed paper, rough and uneven edges, new Ronaldson type, and is illustrated with four beautiful steel engravings, viz.: "The Pride of the Family," "The Bride," "At the Dentist's," "An Irish Patient." I prize it very highly and have enjoyed several leisure hours pouring over its cheery, witty and humorous contents. The series is to consist of twelve volumes, to be issued one volume each month. I recommend it most cordially. It will be a cure for the blues and that tired feeling nearly all doctors have.

---

THE LAW OF MENTAL MEDICINE THE CORRELATION OF THE FACTS OF PSYCHOLOGY AND HISTOLOGY IN THEIR RELATION TO MENTAL THERAPEUTICS. By Thomas Jay Hudson, Ph. D., LL. D., author of the "Law of Psychic Phenomena," "The Divine Pedigree of Man," etc. Third edition. Chicago: A. C. McClurg & Co., 1903. Price, $1.20.

In this work on mental medicine, it has evidently been the effort of Dr. Hudson to place mental therapeutics on a firmer scientific basis, and lift it from that "humbuggery and hoodooism" which were long thought to be the essential factors in its action. He emphasizes the all-important and far-reaching consequences of suggestion, and makes a strong plea for their better use. The book is on the whole very interesting and full of practical points of importance, not only as a curative agent, but also in dealing with the various classes of mankind with which the doctor is in constant contact.

---

PSYCHO-THERAPY IN THE PRACTICE OF MEDICINE AND SURGERY. By Sheldon Leavitt, M. D. Chicago: Garner-Taylor Press, 79 Fifth Avenue, 1903. Price, $1.25.

In setting forth the principles of Psycho-Therapy in relation to medicine, Dr. Leavitt does not make the sweeping declaration that drugs are a thing of the past, but admits that their presence is essential, though he believes that suggestion is the most potent factor in their curative action. In tracing out the history of therapeutics, he calls attention to the service wrought by homeopathy and various other new doctrines which, though themselves not fundamental, have influenced the modern doctor for the best along all lines. He makes plain the fact that curative suggestion does not mean hypnotic trance, but that the principles of cure are based on rational measures. On the question of adoption by the profession at large of the principles of this new science, he warns against the too radical departure from the old methods, at the same time pointing out the undoubted superiority of the new, in many respects. His methods of procedure are clearly set forth, and everything conducive to the gaining of the patient's complete confidence is fully and thoroughly explained.

---

Saunders' Medical Hand-Atlases.—Atlas and Epitome of Human Histology and Microscopic Anatomy. By Privatdocent Dr. J. Sobotta of Wurzburg. Edited, with additions, by G. Carl Huber, M. D., Junior Professor of Anatomy and Histology, and Director of the Histological Laboratory, University of Michigan, Ann Arbor. With 214 colored figures on 80 plates, 68 text-illustrations, and 248 pages of text. Philadelphia and London: W. B. Saunders & Co., 1903. Cloth, $4.50, net.

This work combines an abundance of well-chosen and most accurate illustrations with a concise text, and in such a manner as to make it both atlas and text-book. The great majority of the illustrations have been made from sections prepared from human tissues, and always from fresh and in every respect normal specimens. The colored lithographic plates have been produced with the aid of over thirty colors, and it is evident that particular care was taken to avoid distortion and assume exactness of magnification. The text is as brief as possible; clearness, however, not being sacrificed to brevity. The editor of the English translation has annotated and altered very freely certain portions of the sections on the adenoid tissues, blood and the blood-forming organs, muscular tissues, special sense organs, and peripheral nerve distributions, making these parts conform to the latest advances in the study of these tissues. The work will be found useful as an atlas, text-book, and book of reference for student and practitioner. We strongly recommend it.

---

Lea's Epitome of Medical Epitomes. — Tuley's Epitome of Pediatrics. — A Manual for Students and Practitioners. By Henry Enos Tuley, A. B., M. D., Professor of Obstetrics in the Medical Department of Kentucky University, Louisville, Ky. In one 12mo volume of 266 pages, with 33 engravings. Cloth, $1, net. Lea Brothers & Co., Publishers, Philadelphia and New York, 1903.

Much has been said pro and con regarding epitomization, but the resultant fact remains that when well done it is highly useful.

Professor Tuley's compact work justifies this statement. He considers the whole subject of Pediatrics from the moment of birth to adolescence, including the anatomy, development, care and examination of infants, the therapeutics peculiar to that age, and the feeding of infants and older children, in full detail. He then covers the various diseases systematically and clearly with the necessary directions and prescriptions. This little work may readily be carried in the pocket and consulted at times when larger volumes are inaccessible. In this way the physician may refresh his knowledge and gain practical points when needed. For the benefit of the student, questions are appended at the ends of the chapters, so that he may test his own knowledge. The volume is a fitting representative of the excellent "Medical Epitome Series."

---

The Medical News Pocket Formulary. By E. Quin Thornton, M. D., Assistant Professor of Materia Medica in the Jefferson Medical College, Philadelphia. New (sixth) edition. Leather, wallet shape for the pocket, $1.50, net. Lea Brothers & Co., Philadelphia and New York, 1904.

Professor Thornton has again brought this useful pocket companion thoroughly to date. It is arranged alphabetically and the best therapeutics of the masters is therefore instantly at command in its pages. A peculiar and valuable feature is found in the discriminating annotations. The various stages and complications of disease require treatment adapted to the conditions they present, and rational and successful therapeutics must recognize this fact. In a few clear words Professor Thornton points out the special utility of each of the various prescriptions recommended under the headings of the respective diseases. The book opens with 16 pages of data which the physician will find useful, such as incompatibles, poisons and antidotes and a table of maximum and minimum doses. With this handy volume in his pocket, the practitioner need never feel at a loss for a pertinent suggestion.

---

American Edition of Nothnagel's Practice.—Diseases of the Pancreas, Diseases of the Suprarenal Capsules and Diseases of the Liver. By Dr. L. Aser of Vienna; Dr. E. Neusser of Vienna, and Drs. H. Quincke and G. Hoppe-Seyler of Kiel. The entire volume edited, with additions, by Frederick A. Packard, M. D., late physician to the Pennsylvania and to the Children's Hospitals, Philadelphia; and Reginald H. Fitz, M. D., Hersey Professor of the Theory and Practice of Physic, Harvard University Medical School, Boston. Handsome octavo of 918 pages, illustrated. Philadelphia, New York, London: W. B. Saunders Co., 1903. Cloth, $5, net; half Morocco, $6, net.

This book combines in one volume the sum of our knowledge concerning diseases of the pancreas, the suprarenal capsules, and the liver. Any contribution on these subjects is of great interest to the profession, and these monographs, proceeding from such distinguished investigators, will be found of unusual importance. In the sections on the pancreas and the suprarenals, the numerous experiments upon animals cited will be of the greatest value to the pathologist, the clinician, and the pathologic anatomist, affording an insight into the more deep-seated processes, and offering an opportunity of comparing the disturbances of function produced by morbid conditions experimentally induced, with bedside and autopsy observations. In editing these sections the editor has availed himself of the writings of Korte and Mayo Robson, especially the latter's important treatise on the etiology and treatment of chronic pancreatitis. An editorial addition to the section on the suprarenal capsules which seems especially noteworthy, is the investigations and discoveries on the active principles and therapeutic properties of suprarenal extract.

The excellent article on the liver is as thorough and complete as those on the pancreas and suprarenals. Dr. Packard's careful clinical work, and his interest in the diseases of the liver, mark him as the most suitable person to edit this article. A survey of this work shows numerous critical additions, embodying the very latest contributions, besides expressions of his own views regarding subjects under discussion. He has devoted special care to diagnosis and treatment, including the surgical procedures that have recently found their place in this field. With these numerous editorial additions the articles are brought fully up to date, and have no equal in our language.

---

Practical Points in Nursing.—For Nurses in Private Practice.—With an appendix containing rules for feeding the sick; recipes for invalid food and beverages; weights and measures; dose list; and a full glossary of medical terms and nursing treatment. By Emily A. M. Stoney, late superintendent of the Training School for Nurses, Carney Hospital, South Boston, Mass. Third edition, thoroughly revised. Handsome 12mo of 458 pages, fully illustrated, including eight colored and half-tone plates. Philadelphia, New York, London: W. B. Saunders & Company, 1903. Cloth, $1.75, net.

The continued and increasing popularity of this little volume has placed the publishers under the obligation of keeping it abreast of the times, of making it reflect the latest advances in the progressive profession of nursing. The revision has been extensive, every page showing evidences of careful scrutiny. Considerable portions of the work have been either amended, modified, or amplified in accordance with the progressive spirit of medicine and its indispensable handmaid, nursing. The sections treating of certain diseases, especially the infectious diseases, as well as the treatment of the common poisonings, have been in large part recast and rewritten. By the extensive revision the usefulness of the book has been greatly extended and its trustworthiness enhanced. There is no doubt that the work in its third revised form, will maintain the popularity justly won by the earlier edition.

---

THE STORY OF NEW ZEALAND. By Professor Frank Parsons, the well-known writer and authority on law, economics and sociology; edited and published by C. F. Taylor, M. D., editor and publisher of *The Medical World,* and of "Equity Series," 1500 Chestnut Street, Philadelphia, Pa. Handsomely bound in cloth, heavy paper, over 170 illustrations, many of which are full page. 836 +xxiv equals 860 pages; price $3.00 net.

The volume is unique in the literature of the world. It is history, biography, psychology and sociology welded into one, lighted with wit and humor, charming as the unfolding of a powerful drama, forceful as clear and vigorous English can make it. Never before has the political, industrial and social history of a people been gathered into one richly illustrated and delightful volume. Never has a writer had a subject more full of hope for the solution of the most pressing problems of this age. Never has the relation between successive increment of popular control over the government and the improvement of industrial conditions been so clearly marked. Never has the curve of progress taken an upward sweep so like the path of a rocket. "From savage cannibalism to the highest civilization in a lifetime, from one of the poorest countries in the world to the richest (per capita) in half a century, from racial war to racial harmony in a generation, from industrial war to industrial peace in a decade, from charity to justice, competition to co-operation, monopoly to diffusion, despotism to democracy; governments by landlords and the money power in their own interest to government by farmers and workingmen in the interest of all as the outcome of a great election, is certainly a record of change in condition and policy, which for quantity, quality and speed of progress is without a parallel."

THE CARE OF THE BABY.—A MANUAL FOR MOTHERS AND NURSES, containing practical directions for the management of infancy and childhood in health and in disease. By J. P. Crozer Griffith, M. D., clinical professor of Diseases of Children in the Hospital of the University of Pennsylvania; physician to the Children's Hospital, Philadelphia. Third edition, thoroughly revised. Handsome 12 mo. volume of 436 pages, fully illustrated. Philadelphia, New York, London: W. B. Saunders & Company, 1903. Cloth, $1.50, net.

Dr. Griffith's manual on the Care of the Baby is without question the best work on the subject we have seen. The fact of a third edition being called for within such a short time, is sufficient evidence of its popularity. In preparing this edition every part of the book has been carefully revised and brought fully in accord with the latest advances in the subject. Several new recipes have been included in the appendix, making this excellent part of the work even more complete than ever. A large number of new illustrations have been added, greatly increasing the value of the book.

This is a text-book at the University of Texas Medical College, Galveston.

